# Supplementary material for: Toll signalling controls intestinal regeneration in Drosophila
Source: Development. 2026 Jan 19;153(2):dev204794. doi: 10.1242/dev.204794 (PMC12863300; doi:10.1242/dev.204794)
Supplement: Supplementary information [file develop-153-204794-s1.pdf]

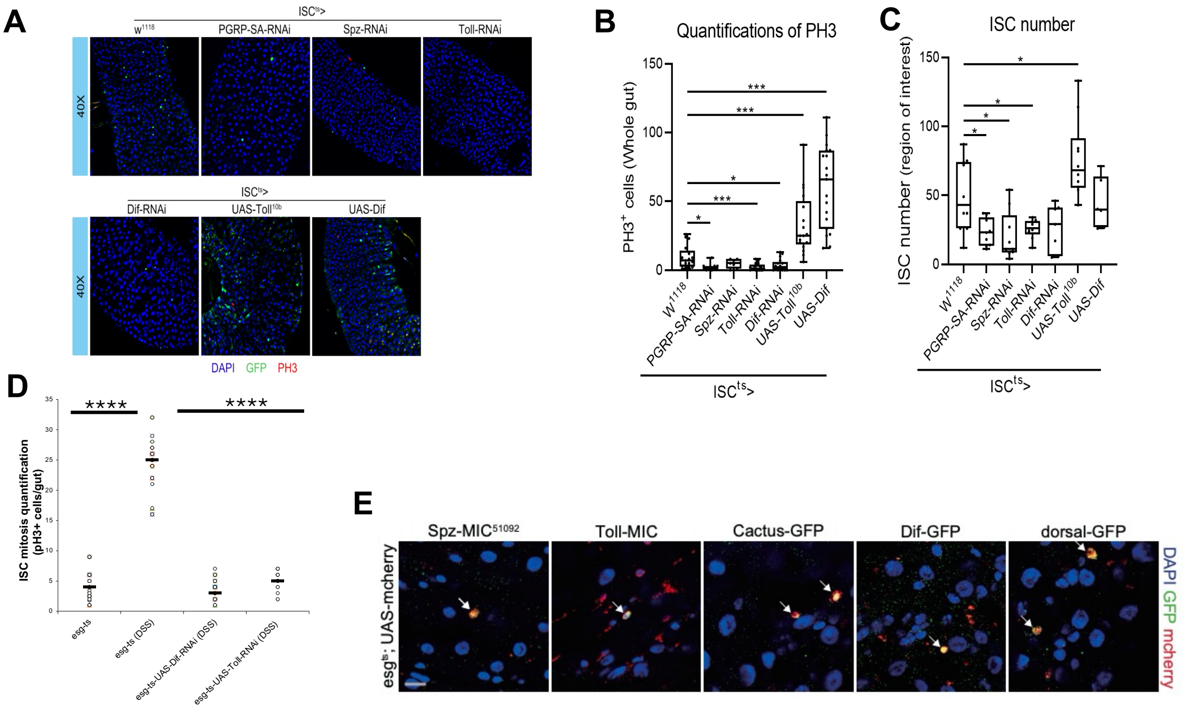

Fig. S1. The Toll pathway is required in intestinal progenitors for the preservation of ISC numbers at homeostatic conditions and after DSS treatment. (A) Merged images of midguts where components of the Toll pathway were depleted (RNAi) or activated (UAS) in ISCs (ISC^{ts}) compared to the control genetic background (*w^{1118}*). GFP (green) indicates the ISCs, DAPI stains all nuclei (blue), and pH3 (red) marks the mitotic cells. **(B)** Quantification of pH3-positive cells in whole guts of flies with treatments as denoted in A (n=20). **(C)** Quantifications of ISCs in whole guts (n=20) with treatments as denoted in A. The Mann-Witney test was used for comparisons between genotypes (*p<0.1, ***p<0.001). **(D)** Quantification of pH3-positive cells per adult midgut of the indicated genotypes with or without DSS treatment at 29℃ for 1 day (after 10 days of normal food). **(E)** Representative midgut images of flies with indicated genotypes expressing *spz*, *Toll*, *cact-GFP*, *Dif-GFP* or *dorsal-GFP* (green) at 29℃ for 10 days. Nuclei (blue), *esg>mcherry* (red). The white arrows indicate GFP-positive progenitor cells.

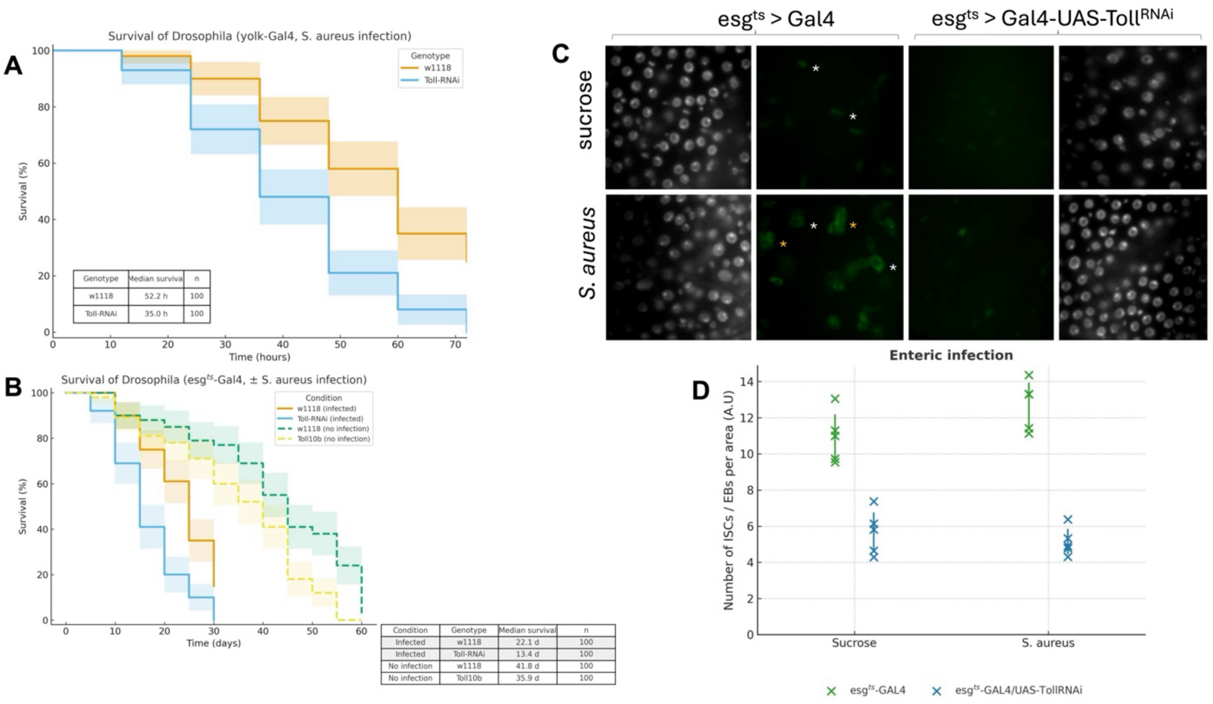

Fig. S2. Toll signalling and host survival following intestinal infection

(A) Survival following systemic infection (injection) of *S.aureus* at 25℃. *w[1118]*, n=108, *Toll-RNAi*, n=102.

(B). Survival following oral infection (feeding) of *S.aureus* at 25℃. *w[1118]*, n=154, *Toll-RNAi*, n=184 or when flies express constitutively active Toll (Toll10b, n=120) in progenitor cells (no infection). In both panels, p-values were extracted by Log-rank tests and the lethal times are represented with 95% confidence interval.

(C). Imaging of intestinal progenitor cells when flies were orally infected with *S. aureus*.

(D). Quantification of intestinal progenitor cells when infected with *S. aureus*. Expansion of intestinal progenitor numbers following infection is Toll-dependent. The Mann-Witney test was used for comparisons between genotypes and treatments. Significant differences ($p<0.001$) were found when control flies infected with *S. aureus* were compared to *esg[ts]/UAS-TollRNAi* infected with the same bacterium as well as when control flies were fed with sucrose were compared to *esg[ts]/UAS-TollRNAi* fed with sucrose.

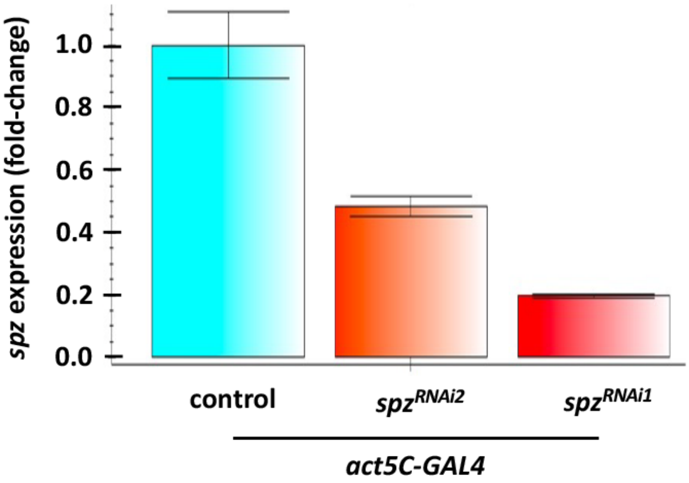

Fig. S3. Effectiveness of *UAS-spz*^{RNAi} lines. The efficiency of two independent RNAi lines targeting *spz* (*spz*^{RNAi1}, BDSC #28538 and *spz*^{RNAi2}, VDRC #105017) was tested by RT-qPCR in L3 larvae by overexpression under the constitutive *act5C-GAL4* driver at 25^{0}C. Each bar corresponds to 3 biological replicates. Both lines silence mRNA expression of *spz* by more than 50%.

Development • Supplementary information

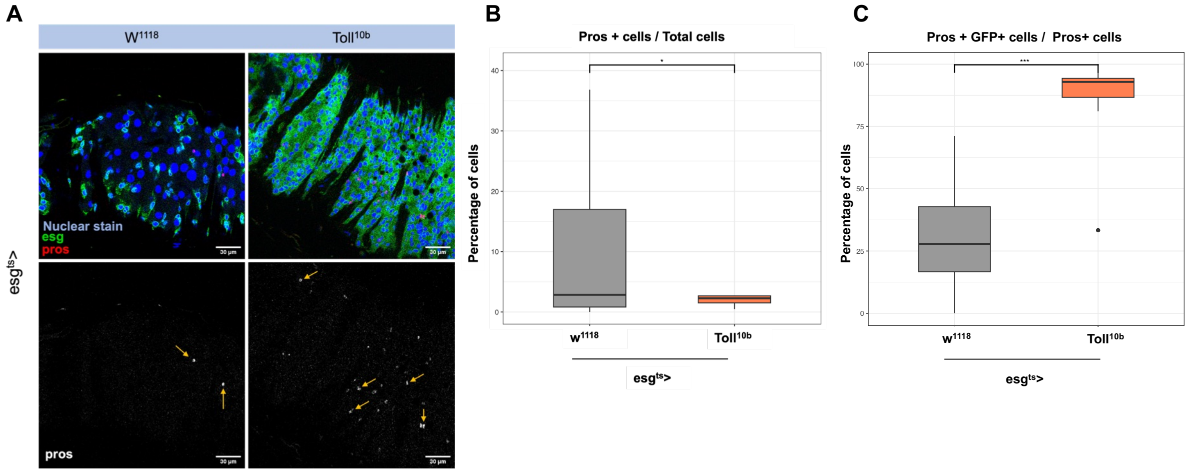

Fig. S4. Activation of Toll signaling in progenitors influences EE cell fates in the posterior midgut. (A) Single confocal frame of posterior midguts (R4 region) of control (*w^{1118}* crossed with *esg^{ts}*) and *esg^{ts}, UAS-$Toll^{10b}$* 10-day adult flies. The progenitors (ISCs and EBs) are GFP+ (green), EEs are stained for Pros (red), and the nuclei are shown in blue (upper panel). The lower panel represents Pros+ cells in white (arrows). Scale bar = 30um. **(B)** Quantification of Pros+ cells (EEs) in the midgut of 10-day old female control (*esg^{ts}* crossed with *w^{1118}*) (n=15) and *esg^{ts}, UAS-$Toll^{10b}$* (n=15) flies. This experiment had three biological replicates. The x-axis represents different genotypes. The y-axis represents the percentage ratio of Pros+ cells (EEs) to the total number of cells in a section of the R4 midgut region. **(C)** Quantification of Pros+ cells (EEs) that are also GFP+ in the midgut region of 10-day old female control (*esg^{ts}* crossed with *w^{1118}*) (n=15) and *esg^{ts}, UAS-$Toll^{10b}$* (n=15) flies. This experiment had three biological replicates. The x-axis represents different genotypes. The y-axis represents the percentage ratio of Pros+ GFP+ cells to the total number of Pros+ cells in a section of the R4 midgut region. Statistics in B and C were performed using the Wilcoxon test (ns = not significant, *p < 0.05, **p<0.01, ***p<0.001, ****p<0.0001).

Development • Supplementary information

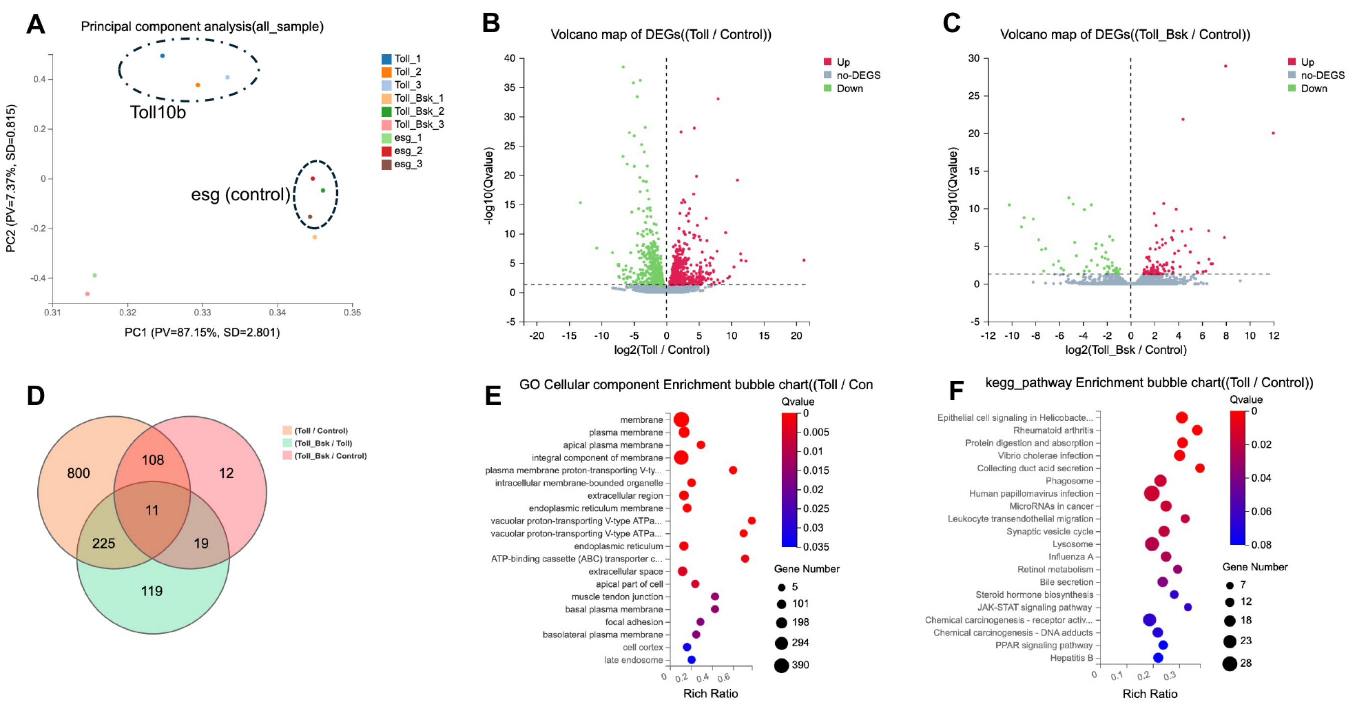

Fig. S5. Transcriptional signatures in Toll^{10b}-mediated intestinal dysplasia. (A) Transcriptomic analysis (bulk RNAseq of whole 20day old guts). Gene expression shown is an an average of three biological repeats. PCA showed that **all** repeats for Toll10b (Toll_1 to Toll_3) and control (esg_1 to esg_3) fell within the statistical range to be able to merge each into one dataset (Toll or control). In contrast only two of the repeats of the Toll_Bsk condition (Toll_Bsk_2 and 3) had that statistical range while Toll_ Bsk_1 was an outlier and was not included in the analysis. **(C)** Differentially expressed genes (DEGs) between esg/Toll^{10b} and esg/control, **(D)** DEGs between esg/Toll_Bsk and esg/control, **(E)** Venn diagram with common DEGs between one, two or all three conditions, **(F)** GO terms for cellular components, **(G)** KEGG pathway enrichment chart. See Table S1 for top 20 DEGs between esg/Toll^{10b} and esg/control and Table S2 for all DEGs across all conditions.

Development • Supplementary information

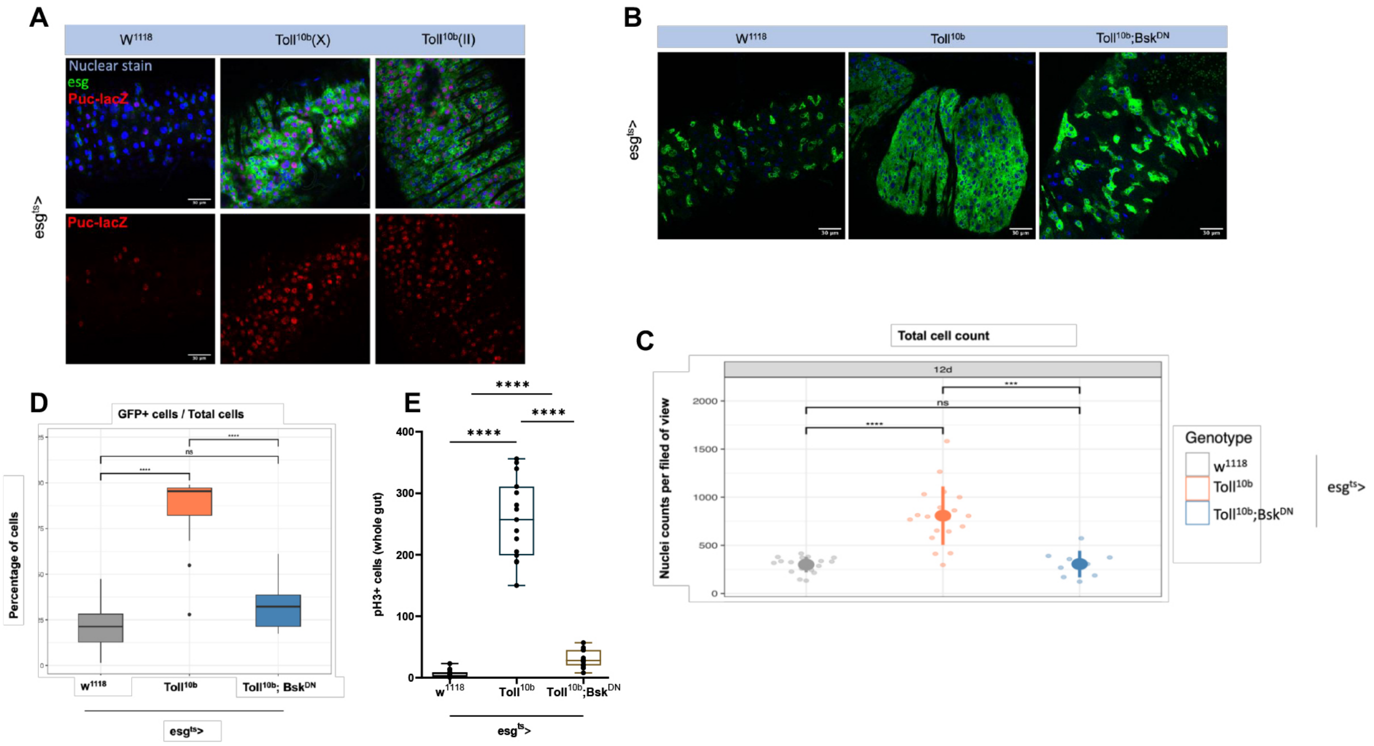

Fig. S6. Effects of Toll activation in intestinal progenitors are mediated by JNK. (A) A *puc* expression reporter (*puc-lacZ*), a transcriptional target of the JNK pathway, exhibited increased activity in $Toll^{10b}$ guts. However, concomitant expression of $Toll^{10b}$ and a dominant negative form of JNK (Bsk^{DN}) suppressed **(B)** the expansion of progenitor cells, **(C)** reducing the total number of cells and **(D)** the ratio of GFP+/total cells as well as **(E)** the number of pH3+ cells. In all cases, *$Toll^{10b}$; bsk^{DN}* flies were statistically indistinguishable from the control. Statistical comparisons were performed with one-way ANOVA (ns = not significant, *$p < 0.05$, **$p<0.01$, ***$p<0.001$, ****$p<0.0001$).

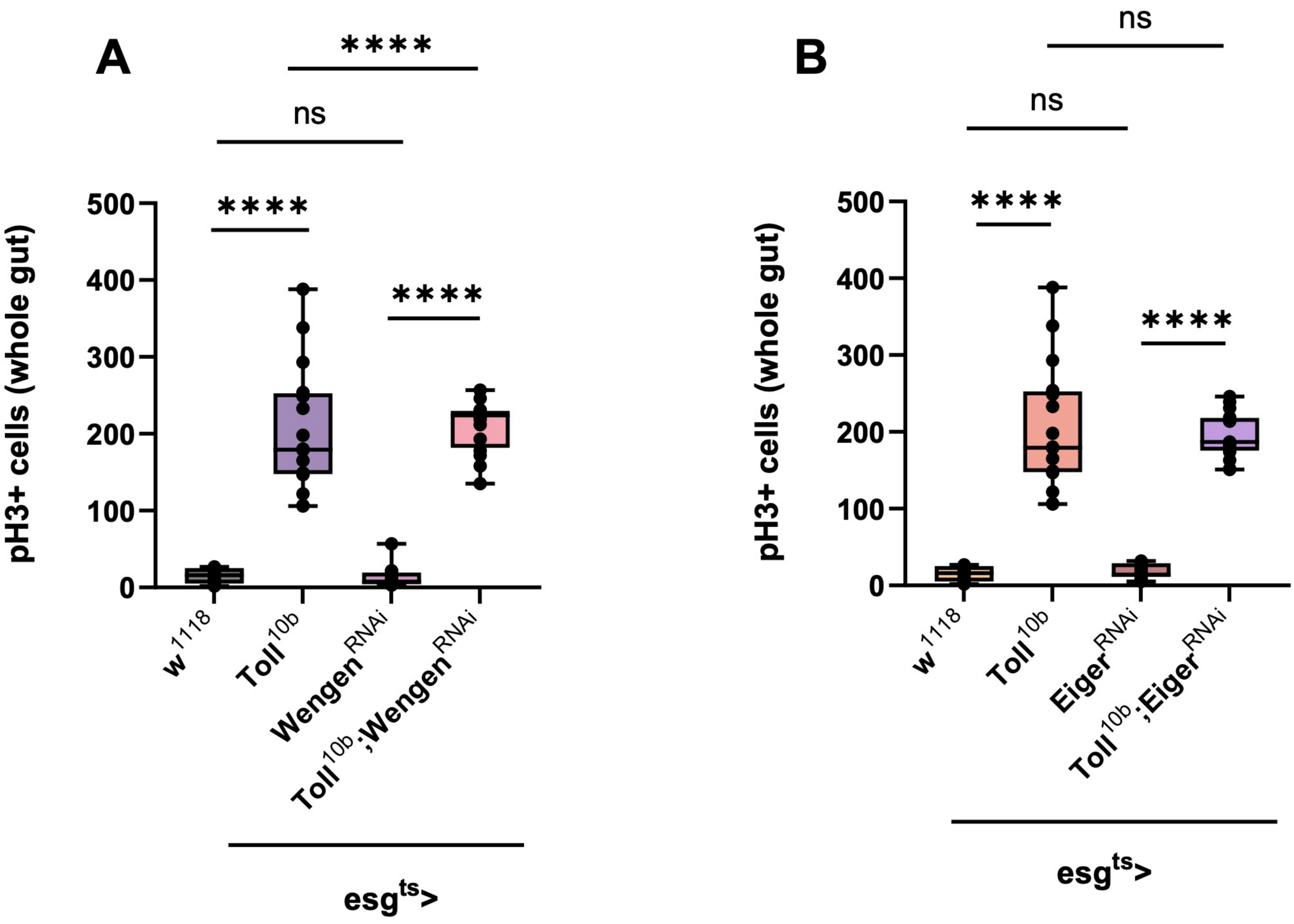

Fig. S7. Wengen-RNAi and Eiger-RNAi do not block Toll^{10b}-mediated expansion of progenitor cells or ISC mitosis. (A) Depletion of the JNK receptor *wengen* in the context of *Toll^{10b}* expression did not reduce ISC mitosis (pH3+ cells). **(B)** Similarly, depletion of the JNK ligand *eiger* did not supress *Toll10b-mediated* ISC mitosis (pH3+ cells). The y-axis represents the total number of pH3+ per midgut. Statistical analysis was performed using the Mann-Whitney test (**p<0.01, ***p<0.001). Controls in both panels are the same as the experiments were done at the same time, side by side.

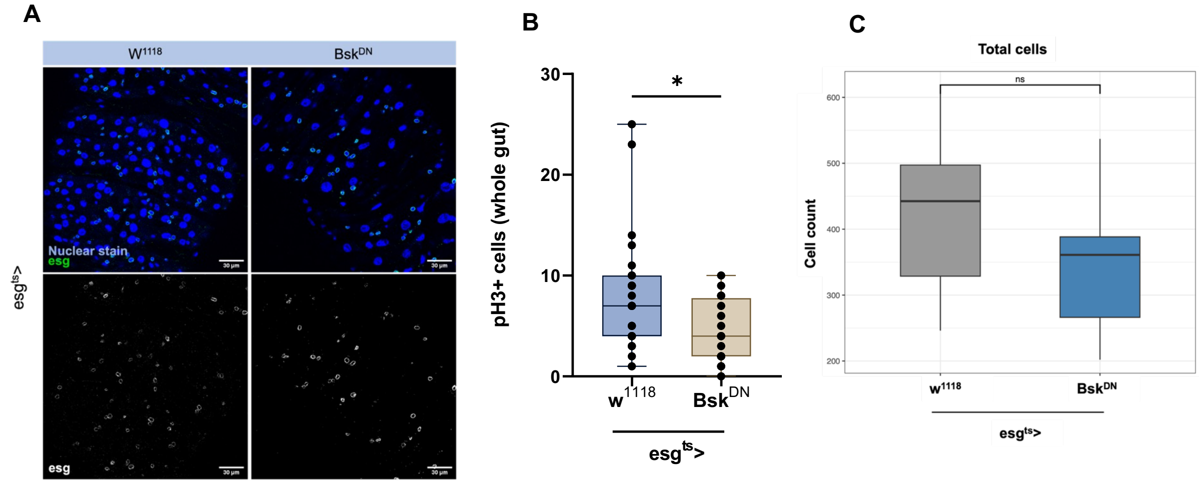

Fig. S8. JNK depletion did not significantly alter epithelial renewal in homeostatic conditions. (A) Single confocal frame of posterior midguts (R4 region) of control (*w*[1118] crossed with *esg*[ts]) and *esg*[ts]*; UAS-bsk*[DN] stained for GFP (green, ISCs and EBs), DAPI (blue, nuclei) (upper panel). The lower panel represents GFP+ cells in white. Scale bar = 30um. **(B)** Quantification of pH3+ cells from whole guts of 10-day old female control (*esg*[ts] crossed with *w*[1118]) (n=23) and *esg*[ts]*; UAS-bsk*[DN] (n=16) flies. This experiment had three replicates. The x-axis represents different genotypes. The y-axis represents the number of pH3+ cells present in the whole gut. Statistically were performed using the Mann-Whitney test (ns = not significant, *p<0.05, **p<0.01, ***p<0.001, ****p<0.0001). **(C)** Quantification of total number of cells present in a midgut region of 10-day old female control (*esg*[ts] crossed with *w*[1118]) (n=30) and *esg*[ts]*; UAS-bsk*[DN] (n=35). Presented are the cumulative results of three independent experiments. Y-axis represents the total number of cells in a section of the R4 midgut region. Statistically were performed using the Wilcoxon test (ns = not significant).

Development • Supplementary information

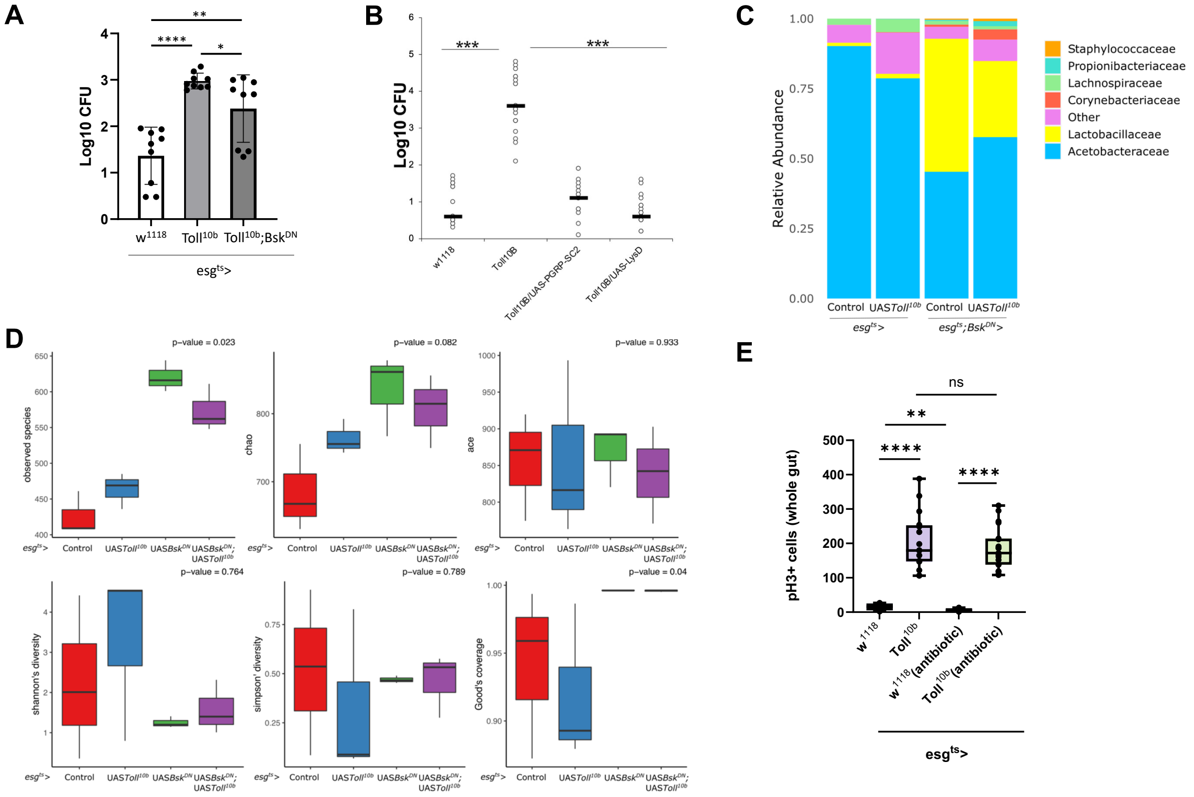

Fig. S9. Toll activation and interaction with intestinal microbiota. (A) Microbial densities in Colony Forming Units (CFUs) on plating 10d old female fly guts from control (*esg^ts^* crossed with *w^1118^*), *esg^ts^ > UAS-Toll^10b^* and *esg^ts^ > UAS-Toll^10b^*; *UAS-Bsk^DN^* (n=15). Student's t-test was used for statistical analysis (ns = not significant, *p<0.05, **p<0.01, ***p<0.001, ****p<0.0001). **(B)** Increase of gut bacterial CFUs in Toll^{10b} was suppressed with co-activation of the amidase PGRP-SC2 or Lys-D (***p<0.001). **(C)** Relative abundance of 16S rRNA gene sequences of intestinal bacterial families. **(C)** Measures of diversity such as the total number of observed bacterial species as well as alpha-diversity indexes such as the Shannon and Simpson indexes showed that the various genotypes were statistically indistinguishable at the level of bacterial family diversity. **(D).** The number of pH3+ cells in conventionally reared and germ-free (antibiotic) *esg^{ts}-GAL4* crossed with *w^{1118}* and *esg^{ts}-GAL4 > UAS-Toll^{1b}* flies. Statistical significance tests were performed using the Mann-Whitney test (**p<0.01, ****p<0.0001). No significant difference (ns) was observed in the number of pH3+ cells in Toll^{10b} flies regardless of the presence of gut bacteria.

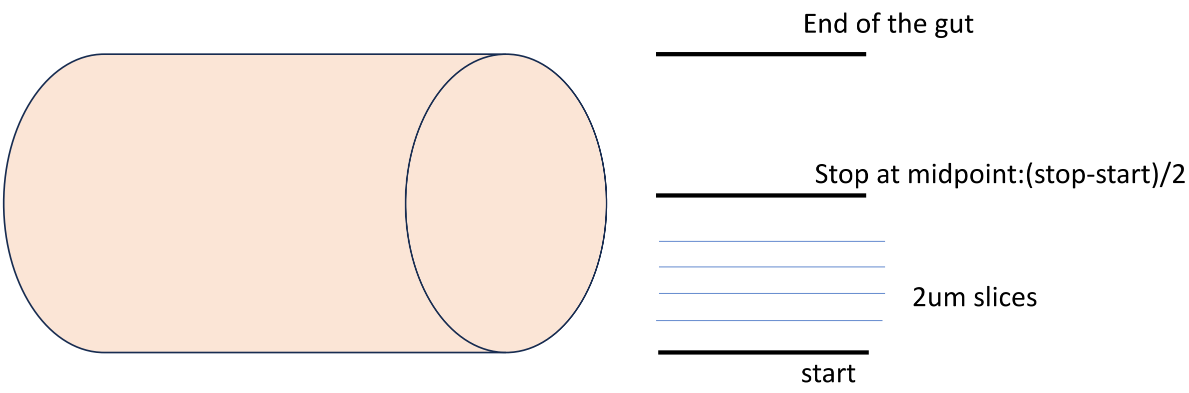

Fig. S10. Representation of z-stacks taken for the gut samples. The start and stop points were taken using the objective. The midpoint of the gut was calculated using: (stop-start)/2. 2um thick z-stacks were taken from start to the midpoint (ending at the lumen).

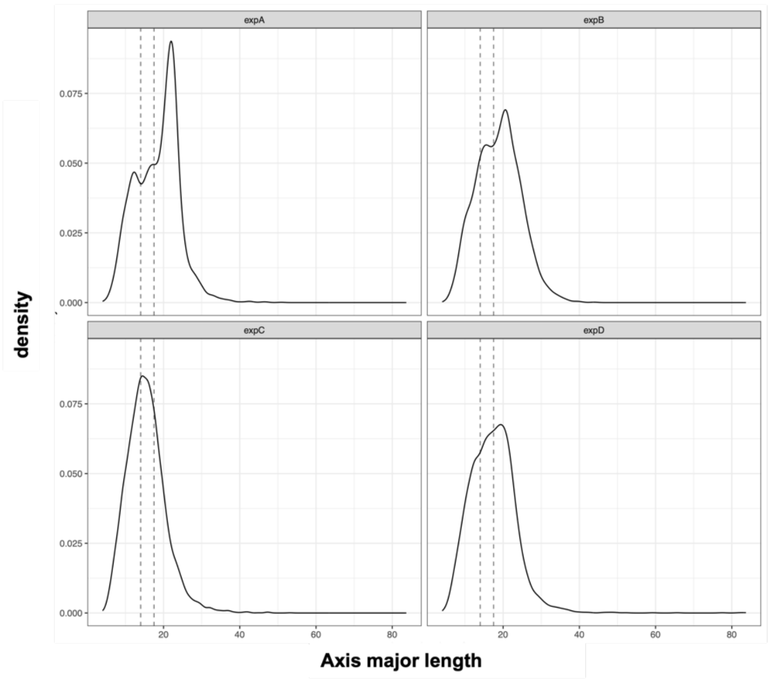

Fig. S11. Histograms for density of nucleus and major axis length.
The figure represents different histograms obtained using different samples from control (*esg*^{ts} crossed with *w*^{1118}) fly midguts. 10-day old flies with stained nuclei were imaged using confocal microscopy and 2um thick z-stacks were obtained. The 3D volumes of the nuclei were quantified, and the major axis length was plotted against density (number of nucleus with that axis length).

Table S1. Top 20 DEGs (*esg*^{ts}/*Toll*^{10B} vs. *esg*^{ts}/*w*^{1118} control) and their functions.

Upregulated gene (Toll10B vs. control)	Function	Suppressed by JNK?	Downregulated gene (Toll10B vs. control)	Function	Alleviated by JNK?
Hdly	Females post-mating	NO	*mwh*	Inhibits actin polymerisation	YES
Dph1	Histidine modification	NO	*LysE*	lysozyme	YES
CG33346	Endonuclease	NO	*LysB*	lysozyme	YES
CG1304	Serine protease	NO	*LysD*	lysozyme	YES
dysf	Transcription factor (cell migration)	NO	*PGRP-SC2*	amidase	NO
Pi3kB2	Kinase (cell proliferation)	NO	*CG9825*	Membrane transporter (amino acids)	YES
Akt	Kinase (cell proliferation)	NO	*Jon99Ci*	Serine protease	YES
Upd3	Cytokine ISC prolf(JAK/STAT)	NO	*Jon65Aii*	Serine protease	YES
Pvf2	VEGF-related (ISC proliferation)	NO	*CG6295*	Lipase	YES
Swim	Wg signalling (secreted signal)	NO	*CG33965*	Lipid transferase	NO
cv-2	Binds to BMP receptors to regulate signalling	NO	*CG6296*	Triacylglycerol lipase	YES
Tsp42Ea	Tetraspanin (cell membrane)	YES	*Cyp4d1*	Cytochrome P450 4d1 (hormone metabolism)	NO
CG18179	Serine protease	YES	*Cyp4d8*	Cytochrome P450 4d8 (hormone metabolism)	NO
Annexin B11	Calcium dependent phospholipid binding (signalling, cell movement)	NO	*CG14219*	Acyltransferase	YES
wun2	Lipid phosphate phosphatase (lipid transport)	YES	*CG14205*	Acyltransferase	YES
CG31077	Chitin binding	YES	*Mdr50*	ABC transporter	YES
axed	Transcription factor (cell/neuron maintenance)	NO	*CG6834*	Myosin binding	YES
Tsp42Em (lbm)	Tetraspanin (cell junctions)	YES	*CG8560*	Carboxypeptidase	YES
Mes2	Transcription factor (ecdysis)	YES	*Srr*	Amino acid catabolism	YES
dAGPS	alkylglycerone-phosphate synthase (lipid metabolism)	YES	*CG9682*	Unknown	YES

Development • Supplementary information

Table S2. Aging-related DEGs (*esg*^{ts}/*Toll*^{10B} vs. *esg*^{ts}/*w*^{1118} control) and their functions.

Available for download at
https://journals.biologists.com/dev/article-lookup/doi/10.1242/dev.204794#supplementary-data

Table S3. Amino acid metabolism DEGs (*esg*^{ts}/*Toll*^{10B} vs. *esg*^{ts}/*w*^{1118} control) and their functions.

Available for download at
https://journals.biologists.com/dev/article-lookup/doi/10.1242/dev.204794#supplementary-data

Table S4. Cancer-related DEGs (*esg*^{ts}/*Toll*^{10B} vs. *esg*^{ts}/*w*^{1118} control) and their functions.

Available for download at
https://journals.biologists.com/dev/article-lookup/doi/10.1242/dev.204794#supplementary-data

Table S5. Carbohydrate metabolism-related DEGs (*esg*^{ts}/*Toll*^{10B} vs. *esg*^{ts}/*w*^{1118} control) and their functions.

Available for download at
https://journals.biologists.com/dev/article-lookup/doi/10.1242/dev.204794#supplementary-data

Table S6. Cell growth and death-related DEGs (*esg*^{ts}/*Toll*^{10B} vs. *esg*^{ts}/*w*^{1118} control) and their functions.

Available for download at
https://journals.biologists.com/dev/article-lookup/doi/10.1242/dev.204794#supplementary-data

Development • Supplementary information

Table S7. Development and regeneration-related DEGs (*esg*^{ts}*/Toll*^{10B} vs. *esg*^{ts}*/w*^{1118} control) and their functions.

Available for download at
https://journals.biologists.com/dev/article-lookup/doi/10.1242/dev.204794#supplementary-data

Table S8. Digestion-related DEGs (*esg*^{ts}*/Toll*^{10B} vs. *esg*^{ts}*/w*^{1118} control) and their functions.

Available for download at
https://journals.biologists.com/dev/article-lookup/doi/10.1242/dev.204794#supplementary-data

Table S9. Endocrine-related DEGs (*esg*^{ts}*/Toll*^{10B} vs. *esg*^{ts}*/w*^{1118} control) and their functions.

Available for download at
https://journals.biologists.com/dev/article-lookup/doi/10.1242/dev.204794#supplementary-data

Table S10. Energy metabolism-related DEGs (*esg*^{ts}*/Toll*^{10B} vs. *esg*^{ts}*/w*^{1118} control) and their functions.

Available for download at
https://journals.biologists.com/dev/article-lookup/doi/10.1242/dev.204794#supplementary-data
